# Improving the quality of palliative and terminal care in the hospital by a network of palliative care nurse champions: the study protocol of the PalTeC-H project

**DOI:** 10.1186/1472-6963-13-115

**Published:** 2013-03-25

**Authors:** Frederika E Witkamp, Lia van Zuylen, Paul J van der Maas, Helma van Dijk, Carin CD van der Rijt, Agnes van der Heide

**Affiliations:** 1 Department of Public Health, Erasmus MC, University Medical Center Rotterdam, Rotterdam, the Netherlands; 2 Department of Medical Oncology, Erasmus MC, University Medical Center Rotterdam, Rotterdam, the Netherlands

**Keywords:** Health services research (MeSH), Nurse (MeSH), Quality of health care (MeSH), Study protocol, Terminal care (MeSH)

## Abstract

**Background:**

The quality of care of patients dying in the hospital is often judged as insufficient. This article describes the protocol of a study to assess the quality of care of the dying patient and the contribution of an intervention targeted on staff nurses of inpatient wards of a large university hospital in the Netherlands.

**Methods/Design:**

We designed a controlled before and after study. The intervention is the establishment of a network for palliative care nurse champions, aiming to improve the quality of hospital end-of-life care. Assessments are performed among bereaved relatives, nurses and physicians on seven wards before and after introduction of the intervention and on 11 control wards where the intervention is not applied. We focus on care provided during the last three days of life, covered in global ratings of the quality of life in the last three days of life and the quality of dying, and various secondary endpoints of treatment and care affecting quality of life and dying.

**Discussion:**

With this study we aim to improve the understanding of and attention for patients’ needs, and the quality of care in the dying phase in the hospital and measure the impact of a quality improvement intervention targeted at nurses.

## Background

Providing end-of-life care in a hospital is challenging, because hospital care is typically focused on prolonging life. Several studies have described the unmet needs of patients dying in hospitals, such as poor symptom control and insufficient communication [1-6]. Gaps in end-of-life care have been identified, e.g. the lack of awareness of approaching death, and shortcomings in healthcare professionals’ knowledge of and skills in palliative care [1-8]. To date research on end-of-life care in hospitals has been mainly descriptive, focusing on the characteristics of care, identifying problems and suggesting possibilities for improvement. In a literature review, Al-Qurainy et al. (2009) proposed improvement strategies: integration of palliative care services in the hospital to enhance caregivers’ attention for the transition of treatment goals; increase of palliative care knowledge among healthcare professionals; and improvement of prognostication, advanced care planning and communication [9]. However, experimental studies on quality improvement interventions in end-of-life care in the hospital are scarce, partly due to methodological challenges in health services research in general and in the field of palliative care in particular. Many results of studies on quality improvement interventions are thus affected by concerns about the validity and reliability of data, due to e.g. limitations of the design, selection bias, inaccurate measurements and confounding [7,10-14]. To evaluate the effects of changes in palliative care structures and processes on patient outcomes, innovative experimental research is needed [7,15-21].

In the Netherlands most in-hospital deaths occur on wards that lack specific palliative care expertise. Innovations to improve the quality of end-of-life care in the hospital have to be disseminated to all these wards and to be integrated in the whole hospital care system [9]. This process of quality improvement seems to be comparable to innovations in other fields of hospital health care, such as infection prevention, and tissue and wound care. To address these problems, networks of specialized nurses, such as infection control link nurses have been implemented in many hospitals. Only few studies have evaluated the effects of these link nurses’ networks, but the results were promising [22-26]. Some work has been done on networks of palliative care nurse champions in the UK, and evaluations indicated that champions’ self reported knowledge on palliative care, and confidence in collaborative working had increased [27-30]. In the Netherlands a few hospitals have recently started such a network. The empowerment of hospital nurses in being an ambassador of palliative and end-of-life care and in the dissemination of palliative care knowledge and skills could contribute to the quality of care of patients dying in the hospital [31-33]. This implies the translation of knowledge and skills from palliative care experts via the network of nurse champions to the wards. The translation of knowledge is a complex process, partly because various types of knowledge (e.g. explicit and tacit) are involved, and it has to be received by various persons in various contexts. Therefore such translation requires various teaching skills [34]. Transfer of theoretical knowledge by experts to ward nurses easily conflicts with nurses’ daily practice; nurses may decide that it is irrelevant, or that implementation is impossible [35,36]. A network of ward based nurse champions, as intermediates between palliative care experts and ward nurses, is expected to improve the results of education. Nurse champions are probably more dedicated to palliative care than other nurses and have more insight in the culture and processes on their wards than the experts. It is thus probably easier to adapt the educational programme to prior knowledge, beliefs, attitudes, and educational needs of the champions than of all nurses on all the wards, and the content of the programme will easier be experienced as meaningful when champions can connect it to the context in which they work [34,37].

Education and training of health care professionals will only be successful when it leads to improved practice and decision making [38,39]. In palliative care this means timely identification of patients in need of palliative and end-of life care, timely referral to palliative care experts, collaboration between patients, family and the medical team, the use of guidelines and appropriate working procedures, and knowledge of palliative care and symptom management on the clinical wards [8,40-43]. Being a resource and role model for their colleagues, nurse champions can contribute to improved quality of palliative care, when they have sufficient clinical experience, improved knowledge of palliative care, improved teaching capacities, and acquired authority towards managers and colleagues. [25,28]. Still, rigorous evaluation of the effects of nurse champions on the outcome of care is necessary. In this article we describe the study protocol of the PalTeC-H project: a study on understanding and improving Palliative and Terminal Care in the Hospital by implementing a palliative care network of nurse champions.

## Methods

### Objectives

Objectives of this study are (1) to explore and understand the impact of the quality of care on the quality of life at the end of life and the quality of dying in a hospital and (2) to investigate the contribution of a quality improvement intervention which consists of the implementation of a network of palliative care nurse champions. We define end-of-life care as care provided during the last three days of life (at most). We hypothesize the implementation of the network to result in more attention for palliative care, in improved and timely recognition of patients’ palliative care needs, in more involvement of palliative care experts and, eventually, in improved quality of life during the last three days of life, improved quality of dying and increased satisfaction of bereaved relatives.

### The intervention

The intervention consists of the establishment of a palliative care network of nurse champions which indirectly affects care by three main components: education, knowledge dissemination and support, plus several organizational elements (Table 1). On intervention wards two staff nurses are appointed to be palliative care nurse champions – further referred to as champions. Together they form the palliative care network coordinated by the multidisciplinary consultation team for pain and palliative care. Champions participate in monthly educational meetings of the network and in a targeted education programme of two days annually. The education programme includes palliative care knowledge and skills as well as organizational knowledge and skills, e.g. on planning dissemination of knowledge, in order to teach the champions to be an ambassador of palliative care on the wards and a role model for their colleagues. The educational strategy is based on the principles of constructivist learning and includes multiple approaches [37]. A senior nurse consultant, member of the multidisciplinary consultation team, is assigned to be the network coordinator, supported by the medical oncologist of the team. This network coordinator facilitates the learning process of champions, by organizing meetings and education programmes, and supporting champions individually in their development and in performing activities. The monthly meetings stimulate the incremental grow of knowledge. Working and learning in a network throughout the hospital give champions the opportunity to share knowledge and learn from others’ experiences, and to capture knowledge from outside their own working environment [23,28,34].

**Table 1:** The intervention

**Phase**	**Activities**	**Method**
Preparation	Ward selection	Registration multidisciplinary consultation team
		Literature review
		Consent of 7 ward managers
	Organization	Selection of 14 palliative care nurse champions
		Appointment of a coordinator
		Development and planning network and education programme
Introduction and follow-up	Composition network	Contact coordinator, ward manager and nurse champions on intervention wards
	Meetings	Every month 90 minutes (9 meetings per year)
	Education	Targeted education programme 2 days yearly and at every network meeting
	Mission/champions’ activities	Dissemination of knowledge (lessons, bedside teaching, being a resource)
		Planned activities on each ward
		Promotion of consulting multidisciplinary consultation team on pain and palliative care
		Implementation of problem based care pathways or protocols on wards
		Acting as a role model
	Support	Coaching nurse champions in plans and activities
		Information in organizational journal, information in newsletters
		Discuss compliance with unit managers

Champions need to identify gaps in knowledge on and quality of palliative care on their ward and to raise health care givers’ awareness on patients’ palliative care needs. They have to organize educational activities, implement protocols on palliative and terminal care, and evaluate these activities at the end of each year.

Assuming that 14 champions each spend eight hours per month on network activities, and that the coordinator spends 24 hours per month, the intervention costs are estimated at € 50.000 per year.

### Study population

All wards in a large general university hospital in the Netherlands participate in this study, including a specialized unit for palliative cancer care, but excluding the department of psychiatry and the Intensive Care departments.

We collect data on adult patients who died at one of the 18 participating wards after having been admitted at least 6 hours prior to death.

## Design

We designed a controlled before and after study with three phases: 1) pre-intervention phase (16 months); 2) phase in which the intervention is introduced (5 months); and 3) post-intervention phase (16 months). The intervention, i.e. the appointment of two champions joining the network, is introduced in seven wards that regularly admit cancer patients or patients with other chronic and life threatening diseases, such as chronic cardiac diseases and COPD. Although there is not much evidence on the time needed to effectively disseminate expertise and knowledge into clinical practice [31-33], we decided that the introduction phase lasts five months, as a run-up period to generate gradual changes in champions’ behavior [16,38]. In the 11 wards where the intervention is not introduced, the same measurements are performed to control for changes that are not due to the intervention, for example changes in hospital policy (Table 2). These control wards are expected to have a similar number of deaths as the intervention wards.

**Table 2:** Participating wards

**Intervention group**	**Control group**
Cardiology	Haematology
Ear Nose Throat surgery	Internal medicine - gastro intestinal diseases
Gastro-intestinal surgery	Internal medicine – renal diseases
Gynaecology and urology	Neurology
Internal medicine – infectious diseases and endocrinology	Neurosurgery and brain surgery
Lung diseases	Liver and kidney transplant and vascular surgery
Medical oncology and geriatrics	Orthopaedics
	Plastic surgery and dermatology
	Medical Oncology - palliative care
	Trauma surgery
	Thorax surgery

### Endpoints

Primary endpoints to reflect the outcomes of care of the dying are global assessments of patients’ quality of life during the last three days of life and patients’ quality of dying on a 0–10 numeric rating scale, comparable to the global ratings in the Quality of Dying and Death questionnaire [44]. The quality of dying has been suggested to encompass seven domains: physical, psychological, social and spiritual experiences, the nature of health care, life closure and death preparation, and the circumstances of death [45]. Secondary endpoints therefore include symptoms, recognition of approaching death, satisfaction of bereaved relatives with health care (e.g. communication, decision making and care) and presence of relatives at the moment of death. Changes in the process of care, such as nursing interventions, treatment goals and the number of referrals to the multidisciplinary consultation team are also secondary endpoints (Table 3).

**Table 3:** Endpoints

**Quality of life during the last 3 days of life and Quality of dying**	**Process of care**	**Satisfaction with health care in the last 3 days of life**
*Quality of life*: Perceptions by relatives and health care providers of quality of life during last 3 days of life: Global rate (0–10)	*Technical process* Appropriate use of nursing interventions Changes in treatment policy/NTBR	*Patient satisfaction with care:* Perceptions by relatives: Preferences honoured regarding way of dying Satisfaction with:
Physical comfort Psychological well-being Social functioning and well-being Spiritual well-being, being in peace	Symptom management Recognition of imminent death Referrals to multidisciplinary consultation team	- technical process
		- decision making process
		- interpersonal and communication style
*Quality of dying of patient* Perceptions by family and health are providers of quality of dying of patient: Global rate (0–10)		*Relatives’ satisfaction with care* Satisfaction with:
		- technical process
Life closure and death preparation Circumstances of death		- decision making process
		- timeliness and usefulness of information and counselling
		- interpersonal and communication style
*Quality of life of family* Health status Grief resolution		- extent to which patient/family preferences honoured
		- extent to which opportunities provided to patient to complete life meaningfully
		- present at patients’ death

Derived and adapted from Stewart et al. (1999) Conceptual model of factors affecting quality and length of life of dying patients and their families.

In addition, we assess champion nurses’ knowledge on palliative care before and after the intervention and monitor the developing process of the network.

### Data collection

On every participating ward, one or two nurses are assigned to distribute questionnaires to a nurse and a physician involved in each dying patient’s care, within one week after the patient has deceased. Completed questionnaires are sent to the principal investigator (FEW). Three months after a patients’ death a relative is sent a written invitation to complete a questionnaire. In case of non-response this invitation is resent after one month. Data on patient and care characteristics such as diagnosis and do not resuscitate agreements are derived from the patient record, when not available from physicians.

We use three different questionnaires: for physicians (35 items), nurses (55 items) and bereaved relatives (94 items). The questionnaires were developed by a group of experts and criticized by a representative of the hospital patients’ council. Then they were tested on relevance and face validity among members of all targeted groups, and piloted in the first 30 cases. Bereaved relatives are asked to answer questions as patients’ proxy and as unit of care themselves.

Champions’ knowledge and opinions are assessed using the Rotterdam MOVE2PC questionnaire, developed and validated for use among general nurses by our research team (publication in manuscript; FEW, LZ, CR, AH). The network process is investigated by counting the champions’ presence at network meetings and education programmes, assessing their activities on the wards, and assessing the coaching activities of the coordinator.

### Data analysis

To address the first objective, i.e. to explore and understand the impact of the quality of care on the quality of life at the end of life and the quality of dying in the hospital, we will analyze primary and secondary endpoints, their interrelatedness, and possible determinants. We will use data from all participating wards during the pre-intervention phase and the intervention-introduction phase (21 months). To address the second objective, i.e. to investigate the influence of the network of nurse champions, we will compare primary and secondary endpoints between the pre- and post-intervention phase (2 x 16 months). Significant changes in the intervention group that are not found in the control group will be interpreted as differences due to the intervention. To measure a difference of one unit on a 0–10 numeric rating scale for global quality of life during the last three days and global quality of dying between the pre-intervention and post-intervention measurement (phase 1 and phase 3), with an assumed standard deviation of 2.5, we need data on 400 patients: 100 patients before as well as after the intervention on both the intervention and the control wards (Lehr’s formula 16/(1/2.5)^2^ = 100) [46]. Expecting a participation rate of 50% among nurses, physicians and relatives we aim to include 400 cases in the pre-intervention phase and 400 in the post-intervention phase.

Data will be analyzed using descriptive analyses, univariate and multivariate regression analyses, t-tests, ANOVA and Chi square tests.

### Ethical considerations

Approval for this study was given by the Medical Ethical Research Committee of the Erasmus MC. The intervention was assigned to seven wards and randomisation of individual patients was not needed. According to the Dutch legislation informed consent was not required because data is gathered after patients’ death and the study involves no more than minimal risk to the participants.

## Discussion

### Measurement of quality of end-of-life care

The multidimensionality and evolution of care at the end of life have been subject of many studies in the last decades [19,44,47-51]. Quality of life, quality of dying and quality of care are overlapping constructs but can be distinguished [45,52]. Quality of life (at the end of life) involves physical, psychological, social and spiritual experiences, and quality of dying additionally includes the domains of nature of health care, life closure and death preparation, and the circumstances of death [45]. Quality of care at the end of life addresses the extent to which these domains are affected by health care.

We study the quality of care of dying patients and their families, as suggested by Stewart et al. [47]. Stewart’s model suggests that health care structures and processes, such as organization, physical environment, communication and decision making, as well as individual patient factors, e.g. diagnosis, psychological characteristics and religious background, determine the quality of dying. According to this model we study to which extent the structure and process of care and patient factors affect the quality of life during the last days of life and quality of dying in the hospital [45,47].

In consequence of the uncertainty of prognostication, ethical concerns and methodological considerations of prospective measurement in dying patients [53,54], we perform retrospective assessments. We invite relatives to participate 3–4 months after the patient's death and incorporate overlap in items asked to relatives and healthcare providers, to address potential recall bias and differences in the reporting of subjective states, such as pain and anxiety, between patients, relatives and healthcare providers [21,55].

A literature search for instruments investigating different aspects of end-of-life care showed that quality of life instruments do not capture experiences unique to the dying process and focus on physical domains mainly [52,56,57]. In 2008 and 2010, reviews were published on quality of life instruments for use in palliative care [56], quality of dying instruments [58] and instruments for the assessment of care of the dying, [57,59] respectively. It was concluded that the QODD, a measure of Quality of Dying and Death developed by Curtis et al. (2002) is the best tested measure of quality of dying to date, although the developers themselves judged it to be suboptimal [44]. The QODD did not meet our goals precisely, because of e.g. the extent of assessing symptoms at the end of life (two physical symptoms only) and the timeframe of reference (one week to one month before death). In addition, it has not been used as a self-completion questionnaire by relatives [57]. More recently Mayland (2011) published on the ECHO-D questionnaire (Evaluating Care and Health Outcomes – for the Dying), developed to evaluate the impact of the Liverpool Care Pathway for the Dying Patient among bereaved relatives [59]. We specifically aim to investigate preferences and experiences in the last three days of life, the inter-relationship of the different domains of quality of life during the last days of life and quality of dying, and their association with bereaved relatives’ overall satisfaction with the quality of care [21,58,60]. Therefore, we developed three new questionnaires, taking into account the content of previous questionnaires, to include the perspectives of relatives and health care providers.

### Evaluation of the intervention

According to the Medical Research Council (MRC) Framework for Development and Evaluation of RCT’s for Complex Interventions to improve health, the intervention with a network of champions is complex [15,61]. Many ingredients contribute to the effects, such as the individual champions’ knowledge and skills and interdisciplinary collaboration, and “it is not easy precisely to define the “active ingredients” of the intervention” [61]. The performance depends on the activities of the champions in the context of their ward, and the “dose” to which professionals and patients are exposed to the intervention may differ among the wards. A principal element of the intervention is the transfer of knowledge. Knowledge transfer is an interpersonal and cognitive process that can promote change strategies and the utilization of this knowledge [31,32]. Coaching the champions to adapt various approaches for the implementation of their newly acquired knowledge is an important tool in our study, in consequence of the need for simultaneous strategies in health care innovations [33]. To date reported effects of champions’ networks are limited to increased knowledge and confidence of the champions themselves [12,22,23,28,29,35,62]. This study will add information on changes in health carers’ behaviour and eventually on the impact on the quality of life at the end of life, the quality of dying and proxies’ satisfaction with care.

## Conclusions

This study will improve the understanding of and attention for patients’ needs, and the quality of care in the dying phase in the hospital. To our knowledge no studies have investigated this topic to the same extent, from the perspective of both healthcare providers and relatives, or measured the effects of an intervention with nurse champions on the quality of care at the end of life.

## Competing interests

All authors declare that they have no competing interests.

## Authors’ contributions

FEW made substantial contributions to conception and design of the study, is primary investigator in this study and is author of the manuscript. LZ made substantial contributions to conception and design of the study, helped to draft the manuscript, and has revised the manuscript critically for important intellectual content. PM made substantial contributions to conception and design of the study; has revised the manuscript critically for important intellectual content. HD participates in data collection and has revised the manuscript critically for important intellectual content. KR made substantial contributions to conception and design of the study; has revised the manuscript critically for important intellectual content. AH made substantial contributions to conception and design of the study; helped to draft the manuscript, and has revised the manuscript critically for important intellectual content. All authors read and approved the final manuscript.

## Pre-publication history

The pre-publication history for this paper can be accessed here:

http://www.biomedcentral.com/1472-6963/13/115/prepub
